# Notes and News

**Published:** 1900-05-26

**Authors:** 


### Notes and News.

### HOSPITAL MEETINGS.

Hospital for Sick Children, Great Ormond Street.—Annual Meeting at five o'clock p.m., on Wednesday, May 30th.

Hospital for Consumption, Brompton.—Annual Meeting on Thursday, May 31st.

Middlesex Hospital.—Quarterly Court at twelve o'clock noon, on Thursday, May 31st.

Medical Graduates' College and Polyclinic Conversazione.—Four to seven o'clock, on Saturday, May 26th, at 22, Chenies Street.

Medical Graduates' College and Polyclinic.—Dinner at seven o'clock p.m., on Thursday, May 31st, at the Trocadero Restaurant.

A new operating theatre and a children's ward have been opened at the Ashford Cottage Hospital. The children's ward contains seven beds. Further improvements in the shape of a covered verandah on the south side of the building, new drainage, and heating apparatus have been added at a cost of £750.

An experiment is being made by three Italian doctors to rid the town of Tarsari of mosquitos. The method pursued is to saturate the surrounding pools with chlorine and petroleum twice a month. The chlorine is for the destruction of the mosquito, and the petroleum for the larvæ. The expense is calculated at about £40 or £50 per annum for a town with 50,000 inhabitants.

A cottage hospital has been opened at Aberlour to serve the three parishes of Aberlour, Inveraven, and Knockando. It is called after Mr. Fleming, the late donor of the hospital. Mr. Fleming, who died before the completion of the hospital, left ample money in his will for the establishment of the institution, and to start it in working order. Mrs. Fleming paid the legacy duty, that the gift should not be diminished.

Mr. A. F. Hills has presented a cottage hospital to Loughton, in Essex. Mr. Hills has placed the hospital in the hands of Dr. J. Oldfield and the managers of the Hospital of St. Francis. An open-air treatment for medical and surgical cases is to be adopted. Mr. Hills has made a donation of £250 to the hospital, and has stipulated that some beds shall be available for patients of the middle classes who can afford to pay moderate fees.

An isolation hospital has just been opened at Bishop Auckland, to serve part of the County of Durham. It has accommodation for 24 patients, and cost £7,338.

The old cottage hospital at Totnes is to be replaced by a new one, the foundation-stone of which has been laid. It will consist of two general wards, one on each side of the central administration quarters, and a special ward, operating-room, and bath-room. The cost is estimated at about £2,200.

It has been arranged that the Aberdeen Maternity Hospital shall be removed to other quarters. By the advice of the Professor of Midwifery at the Aberdeen Medical School (Professor Stephenson), a building will be acquired within the city, called St. Ninians. It is stated that it is so suitable as to require little alteration—a very unusual case where a building has to be converted into a hospital. The cost is estimated at £4,000.

A new workhouse infirmary has been opened at Basingstoke. The general wards are built as wings to the central block, the first floor of which will be devoted to maternity cases. On the south side of the large wards runs a covered balcony. The accommodation is for about 100 patients, and the cost £10,000. At the opening ceremony a tribute was paid to those who had by care and attention produced a good institution in so economical a manner.

At the meeting of the General Medical Council on Tuesday the President (Sir William Turner) announced that a short Bill had been prepared with the object of obtaining additional powers for penal and disciplinary purposes. He also described the present position of affairs in regard to the Midwives Bill and the reciprocity question, the latter of which had, he said, entered a new phase. In view of the difficulties surrounding this question of reciprocity, the Council would probably desire to express an opinion on the matter during the session.

It is said that an ingenious German has found out that by giving caffein in large doses he can produce palpitation to such an extent as to enable recruits to be declared unfit for military service on medical examination, and that by administering picric acid a sufficiently close imitation of jaundice can be produced to serve the same purpose. A considerable number of recruits having been rejected on account of jaundice and palpitation the authorities smelt a rat, and on inquiries being made it was discovered that quite a trade was being done in getting young fellows off their compulsory service by producing imitations of these diseases.

The Hon. Stephen Coleridge's article in the *Contemporary Review*, entitled "Some London Hospitals and their Published Accounts," has caused a correspondence between that gentleman and the authorities of the Middlesex Hospital. This correspondence Mr. Coleridge has circulated in pamphlet form, and it is proving an excellent advertisement to the Middlesex Hospital, as it is bringing attention to bear upon the splendid work the hospital is doing towards the prevention, cure, and treatment of cancer. One gentleman has sent a second donation to be devoted to cancer investigations, as a direct result, as he states, of the pamphlet in question.

